# Mr. Rolfe, on the Influenza

**Published:** 1803-08-01

**Authors:** Thomas Beddoes

**Affiliations:** Clifton


### Medical and Phyſical Journal.

VOL. X.] AUGUST 1, 1803. [NO. LIV.

*Printed for R. PHILLIPS, by W. Thorne, Red Lion Court, Fleet Street, London.*

### *To the Editors of the Medical and Phyſical Journal.*

GENTLEMEN,

WITH this you have the first part of my promised Collection. It is perhaps only necessary to premise, that my Queries chiefly related to the propagation of the Influenza; to its affinity with common catarrh, pneumonia or low fever; and to the time and manner of its appearance and disappearance.

I should not find it easy to reduce my materials to strict order, even if they were to receive no addition; but I have aimed at some kind of arrangement, by placing those Communications first, which are decided in one way, on the grand question of Contagion; next, the sceptical; and lastly, those which express, more or less positively, an opinion contrary to the first. I fear you will think my packet too bulky for one of your numbers; and yet the two next will probably be larger.

*Clifton, July* 4, 1803. THOMAS BEDDOES.

1. Mr. HANSARD, Bristol, April 12, 1803.

I remember seeing patients in the disease called Influenza of the year 1775; but have seen nothing in the present Epidemic that comes exactly up to the character of the disease of the above year; that was, I believe, generally admitted to be contagious; this, I think, cannot be.

A fine boy, six years old, was seized with symptoms of Influenza; his disorder continued twelve days, after which he recovered. A younger sister and elder brother to the above boy slept every night in the same bed with him, yet both escaped the disease. A stout healthy man, twenty-eight years old, was seized with symptoms of strongly marked Influenza; he continued ill fourteen days; the plan laid down for his recovery was wholly neglected, and a very bad one adopted by his friends: he died. His wife slept every night in the same bed with him, yet she escaped the disease. A similar case with the first I have stated, occurred in my own family, and we are eight in number.

I think the influenza distinguishable from common catarrh.

A few instances have occurred so very complex in their symptoms, that I could not decide to what class they belonged; but generally gave way to the antiphlogistic plan.

There was, on various occasions, an alarming and sudden depression, not often met with in common catarrh, so much so, that even cordials and antiseptics seemed as indispensably necessary as in low and putrid fever.

On the 2d of March I saw the first case, and on the 4th of April the last.

2. Mr. Rolfe, Bristol Dispensary, April 11, 1803.

The disease in question I have from the first considered as epidemic; and on referring to the admission book of the Bristol Dispensary, I find I have admitted from the commencement of the present year to this time, 721 patients, out of which have been 153 well marked cases of catarrh, which are entered accordingly; also from 50 to 60 more, I am now well satisfied, had the same disease, which was entered under the names of peripneumonia notha, tussis, &c. from its partaking of its different variations with which it has been attended.*

Notwithstanding that whole families have been attacked with this disease, I have had no reason to imagine it arose from the sick person, as, in several instances where some part of the family have been kept from the sick person entirely, they have been equally affected; and in no instance have I found the nurses, or those more immediately exposed to its influence, the more generally affected. My own maid-servant was attacked with the complaint, and was confined for several days; and though I have eight in family, (and whilst she was ill nine) yet no one was affected by it but herself.

I will not allow myself competent to judge practically of the influenza, it having raged just before I was in practice.

* From this number of well-marked cases, 127 are cured, 28 relieved and on the books, and 6 died. Those who died, with the exception of one sucking child, were all predisposed to habitual pulmonic affection.

tice. The present epidemic I consider as catarrh, under which I have generally entered it.

As far as I am able to judge of influenza theoretically, I suppose them both the same.

The first well-marked case I observed after it appeared as epidemic was Feb. 18; and the last, which is now on the books, is April 8.

3. Mr. Lee, Bristol, April 7, 1803.

I might have been enabled to speak decisively; but confidence in my ability to judge, hath not kept pace with the opportunities afforded me; and from what I have observed, I am more willing to say that the prevailing disease hath not been contagious, than able to prove such an opinion to be right.

The very partial existence of the disease in families consisting of nine, thirteen, and fifteen persons; one, two, or at most three of whom became affected with the prevailing disease. When the possibility of its being contagious was first mentioned to me by a lady, who with her husband and nine children, servants, &c. resided in the same house, I confidently resisted the proposed separation of the member of the family then affected, from the rest; and in that family one more only hath been attacked with the disease. As I do not read news-papers, I did not at the time I so offered my opinion, know that an awe-striking trumpet from on high was vibrating on the public ear; but since so much discussion hath taken place, I have endeavoured to observe more precisely; and, only, am not more confident in my first opinion, from seeing that men, far above my powers to estimate, either in abilities or acquisitions, differ so essentially upon points by me at least taken upon the most blind and unmistrusting confidence, as proved and undoubted.

I do not think the prevailing disease; that is, such cases of disease, very similar in their type and yielding to the same treatment as have occurred in my limited practice, betwixt the latter end of February and March, deserving of any such specific name as influenza. I think the same disease would occur on the same previous and subsequent state of the atmosphere, with respect to heat, cold, or winds occurring; and I have a perfect recollection of a similar affection being very general in the spring of the year 1798. The disease was unbaptized that year; but flippant Paris hath chattered about La Grippe, and too imitative England must gabble about Influenza. One of the most grave French historians have remarked, that in Paris much is ever done by a name, however given, obtained, or preserved : in England we may remark, much hath been attempted, and some of us may feel that something hath been done by a name.

Where I hesitated as to, or denied the existence of, influenza, I did not seek for diffeiences or distinctions betwixt that and catarrh. To call diseases names, or to break or fritter their various hues into shades, may be safely abandoned to artist-godfathers and godmothers, in that way so that they be not often abusive, or often too nice; and whether the prevailing disease be called catarrh properly or not, I am not, nor care to be at present, Nosologist enough to determine; this is the *arcanum imperii*, the lid of which it would be presumptuous even to meditate to lift up, much less would it be allowed me to peep into.—But if compelled to be decisive, I would not name the disease either catarrh or influenza.

On various occasions there certainly was greater and more sudden depression than is almost ever seen in common catarrh; and the disorder appeared, at various times, in almost all the gradations from pneumonia to low fever. To whom is it unknown, that in the gradations of fever, anxiety and depression assist essentially to form the diagnosis? at least, so I have been taught; and this is one of the few teachings that experience hath yet confirmed.

On the 25th of February I was consulted by my friend the Rev. Mr. Shaw, and who, instead of resorting to little things to try what they would do, as the foolish phrase is, availed himself immediately with the happiest effect of a good dose of the acetated ammonia in solution; two days and the contents of two phials put an end to his disease and inconvenience. The 27th ult. was the last case, but that had been standing nearly a fortnight, and is yet uncured.

I hope the limitations your queries confine me to observe, may not prevent me saying in this place, that the plan of treatment proposed by yourself hath been generally useful, as I verily believe; and in my own practice successful, without exception, in the progress and termination of fifty-five cases.

You will readily believe that what I have thought freely, I could not mean to express offensively; but unaccustomed and unwilling without consideration *jurare verbo magistri*, I have given you my sentiments such as they are; and no other you could require.

4. Mr,

4. Mr. Simpson, Bristol, April 6, 1803.

Robert Simpson does not consider the present prevalent disease, called the Influenza, to be at all communicative from one person to another. Considerable observation in families and persons, have led him to conclude it not contagious. Much hurry of business prevents R. S. from entering further into the other queries.

5. Mr. Griffith, Bristol.

From the observations I have made on the Influenza, I am of opinion that it is not contagious.

My reasons for thinking the Influenza not contagious, are, that, in many instances, I have observed it did not attack those persons who from particular circumstances were not exposed to the common atmosphere, while individuals of the same family, who were in the habit of being out in the air, were attacked with the disease.

2dly. Although a free communication was kept up with persons labouring under the disease, and those who were not subject to exposure in the air, the latter had not the slightest appearance of it.

I think the influenza differs from a common catarrh in some particular symptoms, i. e. the pain in the limbs, which has more or less attended it, and the loss of strength and depression of the animal spirits.

On the first appearance of the influenza, I considered it as a catarrh brought on by the vicissitudes of the weather, but at the same time observed that it was attended with some unusual symptoms.

On many occasions there was greater and more sudden depression than is usually seen in common catarrh; the disease sometimes put on the appearance of pneumonia; in the course of two or three days the inflammatory symptoms considerably abated, and not unfrequently left the patient in a state resembling that of low fever.

The first case of influenza which I observed to differ from a common catarrh, was on the 2d of March, and the last case was on the 10th of April.

6. Mr. Short, Bristol, July 5, 1803.

From every observation, I have been induced to consider the influenza as not contagious.

From several cases of this disease where only one person in a family of seven have been affected (although no seclusion had been observed by way of caution), and in houses consisting of eleven inhabitants, where two have been seized only. I have also attended in cases where the husband has been affected with the influenza, whilst the wife has escaped, although a constant attendant, and as it may be said, breathing the same air; and, vice versa, where the wife has had the disease and the husband not; and should suppose these instances have not been rare.

I have generally found the febrile symptoms in catarrh less violent, likewise more gradual and slow in their effects than influenza.

Perhaps, had not influenza been an epidemical or prevalent disease, one might have been led to suppose these attacks in some instances a species of catarrh, more rapid in its progress from the season of the year; as we generally find inflammatory diseases more acute and violent at this season of the year, than in the other parts of it.

In all cases of influenza, which came under my attention, the symptoms were more rapid and violent than I had ever witnessed in common catarrh; but in respect to that variety of the disease, I cannot say I had remarked it.

On the 12th of March, I was called to visit the first patient, and have not witnessed any fresh attack of this disease since 30 ultimo.

### 7. Mr. Southall, Bristol.

From the observations I have been enabled to make in my own practice, together with the information I have obtained, both by medical and common inquiry, I am of opinion the influenza is not contagious.

Notwithstanding in my own house my wife was most violently attacked with the influenza, together with my two children, a young lady and three servants, I and a gentleman who assists me have escaped; in addition to which, I have attended several severely afflicted, and observed no precaution whatever. These observations extend still farther; where I have attended, in repeated instances, one, two, three, or more in a family have been seized with it, and the rest have escaped; particularly at a female boarding school, where I saw only one who had the influenza, and that to an alarming degree.

I think the influenza distinguishable from a common catarrh, inasmuch as the symptoms are, in general, more violent, painful, and distressing; and also that several attend the first that do not, but in rare instances, accompany the latter; that is to say, a most excruciating pain in the head, so as to draw from the person the expression, that that "*it must surely be splitting in two;*" excessive dejection of spirits, extreme pain in the back and extremities, accompanied with rigor and fever, more than is felt in catarrh.

The diseases are so distinguishable, that I believe the first case I had I remember to have declared it, in my opinion, to be the influenza, and that too before I even knew from medical men or others, that such a disease had taken place; and this declaration resulted from the violence of the symptoms, together with a recollection of the influenza when, prior to this, it was so universal. Under my subsequent observations, I have uniformly found it so distinct from a common catarrh as, in my own mind, in no single instance, to have confounded one with the other.

As I have before remarked, the depression has been excessive, and such as I never have felt or have known others feel in common catarrh; indeed, had not the attack been so sudden, and accompanied with such symptoms as attend the influenza, I should have been led from that circumstance, in some instances, to have suspected approaching derangement of intellect. As to pneumonia, I did not remark any decided tendency to it but in one case, where I was nearly tempted to employ the lancet; but upon delay and more consideration, declined it, and the patient perfectly recovered by nearly the common treatment. With respect to the gradations from pneumonia to low fever, they have not come under my view, because when the inflammatory symptoms have been conquered, no farther medical treatment seemed absolutely necessary.

I saw the first case on March the 7th; and the last, on April the 3d.

8. Mr. Smith, Bideford, June 29, 1803.

Queries by Dr. Beddoes.

1. When did the influenza appear and disappear with you?

2. Was its date different in remote places within your reach?

3. After being general, did it occur for some time in single instances?

4. Did it ever seem to pass from person to person?

5. If so, is it likely that clothes or fomites conveyed it in any case?

Answers by W. Smith.

1. The first well-marked case was the 30th of March, 1803; the last, the first week in May.

2. I was informed (I did not see it) that it prevailed very generally at Hartland, fourteen miles W. of Biddeford, and at Clovelly, ten miles N. W. of Biddeford, at least a fortnight sooner.

3. The disease was peculiarly mild in this town; if it might ever have been said to be general, its symptoms were too slight to require medical attention, excepting in solitary instances.

4. As far as I could discover, never; on the contrary, when several in one house were affected, it seemed as if the power of the disease produced the diseased excitement at the same time; nor could I discover that contact, clothes, or sleeping in the same beds or rooms, increased the number of the sick.

5. Answered as above.

The first family I attended was a potter's, four miles east of Bideford, situated upon a peninsula formed by the conflux of the two rivers Tane and Torridge. The house is solitary, nor could I discover that its inhabitants had suffered any communication with others from whom the disease might be derived within forty-eight hours. The whole house felt its influence.

Bideford and its environs are peculiarly happy in resisting the influence of prevailing epidemics; the experience of nineteen years has strongly illustrated this fact: whilst particularly the autumnal epidemics are making severe ravages within three or four miles of us, we have only a few solitary instances. What is remarkable, before the influenza had run half its career, the scarlatina anginosa became very frequent among our children, and, contrary to the above assertion, became the most general epidemic I have ever seen in this town; it remained also some time after it, but is now gone.

The duration of the influenza was from three to five days, excepting only when symptoms of pneumonia were excited, and the lungs previously diseased. I found anti monials in any stage, and almost in any dose, always exasperated the symptoms, inducing diarrhœa, vomiting, &c. and never producing the desired determination to the skin.

9. Mr.

9. Mr. Davies, Bishop's Castle, May 31, 1803.

The influenza began in this town about the middle of March, and continued full a month. In the neighbourhood, it began full a fortnight later, and is not quite over yet.

It usually attacked with pain in the limbs, pain or giddiness in the head, sickness with vomiting of bile, cough, fever, the tongue becoming from white to a yellow brown, and a very early and great debility, many people for a week or fortnight previous to the above symptoms being much more disposed to perspire upon using exercise than usual; the above symptoms generally continued from five days to a fortnight, but the cough and affection of the head frequently longer. Emetics, antimonials, and aperitives, with sometimes a blister to the breast, was my plan of treatment. I lost only one old lady of 78, but who had been paralytic for a year past; and I do not think there were above two or three more died in this neighbourhood.

It attacked families in so irregular a manner, that I could not trace its progress to be immediately from one person to another. It sometimes attacked two or three children in the same house within twenty-four hours of each other, but adults at more distant times; many families escaped, some one or two in a house, whilst it went quite through sometimes large families. My idea was, that it was not communicated from one person to another.

10. Mr. James, Wrington, Somersetshire, June 22, 1803.

The influenza, which has been very prevalent here as elsewhere, made its appearance about the 14th of March, and seemed at its greatest height about the 21st, during the northerly winds. Many of my patients attributed their illness to being exposed to those winds, and four in particular in the night time on Broadfield Down and the Mendip Hills. The disease continued till the latter end of June, and appeared to me not to spread from person to person, but to be a catarrhal affection depending upon a particular state of the atmosphere.

11. Mr. Tickell, Bath, April 13, 1803.

I judge from my own observation, that this epidemic is by no means propagated by contagious miasma from one person to another, but that it originated and was occasioned by a peculiar constitution of the air, unfathomable to to human foresight, and impossible to be prevented by any precautions.

In seminaries for young people of both sexes, no attention having been paid to keep the sick from the healthy; great numbers escaped, notwithstanding a constant intercourse with each other; even where two have slept in the same bed, and one had the disease violently, the other did not receive the least degree of infection; and though in some houses the influenza has indiscriminately seized the whole family, many instances have occurred where one or two persons have had the disease severely, and the others have altogether escaped.

No two diseases, certainly, are more decidedly marked and easily distinguishable from each other, than influenza and catarrh; but it is to be observed, that now, as well as in the former epidemic, which bore the same name, in the year 1782, common catarrh then, as well as now, very generally prevailed, and THAT complaint was easily removed by general remedies.

I scarcely remember any instance where, by proper attention, it was not easy to distinguish the prevailing epidemic from common catarrh.

When the disease was acute, as it happened in a variety of instances, though seldom fatal except in old or elderly people, I observed that the attack was sudden, without any previous indisposition; that there was a sense of great internal debility; an uncommon degree of oppression at the præcordia; an intolerable and excruciating pain in the fore part of the head, greatly aggravated by cough; loathing of all animal food, and also particularly of bread; great prostration of strength, and almost incessant cough, with difficulty of breathing; a copious and most abundant expectoration, sometimes, but not often, mixed with a small portion of blood; attended with delirium at night, and at various times, with the gradations above-mentioned.—Having myself experienced the symptoms of this disease, in its most formidable shape, I can speak more decidedly on the subject. It is also to be remarked, that a state of great debility continued long after the removal of all symptoms of disease. The state of the pulse corresponded with the degree of debility induced in the system, and for the most part was quick, low, and small. Blood-letting was rarely admissible; and when directed, was rather with a view of diminishing the action of the vessels, by which the blood was propelled with an undue impetus on the brain, than to relieve any pneumonic symptoms.

Aperients

Aperient medicines always afforded considerable relief. The urine was generally very high, sometimes flame coloured, and often with a very deep tinge, but without sediment, even in the decline of the fever. Sweat appeared to me to be the critical discharge, which relieved the patient in a great degree, and terminated the disease.

As far as I recollect, the first patient I visited in this disease was on the 17th of February, and the last seized with it on the 5th of April.

12. Dr. Hughes, Hereford, June 30, 1803.

In most of the cases, which fell under my notice, of the influenza, it appeared to me to differ from common catarrh, in being attended with a much greater degree of general languor and debility, and in not having so copious a secretion of mucus from the nose and fauces as usually happens in catarrh. The invasion of the disease was also frequently much more sudden, and not often marked by any particular rigor or other febrile symptom.

It appeared to prevail in an equal degree in the country as in the town at the same time, except that low marshy situations seemed to favour its progress and preserve its continuance longer than those on higher ground.

The disease made its appearance early in January, and was at its height, in and about Hereford, towards the middle of February, from which time it appeared gradually to decline.

As far as my observation goes, I am inclined to think it was not contagious; at some places which lay high, the disease scarcely made its appearance, though a constant and daily intercourse was kept up with different infected places. At Ross, I apprehend from inquiry, very few persons were seized with it.

One strong characteristic of the disease, was its frequent recurrence, after a remission and in many cases total cessation of the symptoms. Adults were chiefly the objects of its attack.

13. Mr. Jones, Montgomery, June 21, 1803.

The influenza began about the 14th of March, and ceased towards the latter end of April, a few *indistinct* cases appearing somewhat later.

The first cases I saw, were eastward of the town of Pool, about half way between that place and Shrewsbury; the disease did not become general in the town of Pool till about about a week afterwards, and did not make its appearance at any considerable distance westward of that town till some days later. I have been informed by some intelligent persons in the neighbourhood of Llanidloes, a small town about thirty miles west of Pool, that the disorder made its appearance there about a fortnight later than in Pool, and was considerably milder in its symptoms.

My observation does not by any means confirm the notion of its being infectious or contagious. I have seen it under some of its worst forms affect a part of a family in crowded cottages, whilst others of the family escaped it altogether; nay, I have seen sick and well sleep together in the same bed frequently, and the latter remain free from disorder.

The above observations relate to Pool, where I resided at the times above mentioned; I think the neighbourhood of Montgomery felt less of the effects of the disorder than that of Pool. I have not seen or heard of a fatal instance any where near either place; the most threatening cases I saw, were some in which there came on symptoms of violent inflammation of the trachea, but which yielded to blood-letting and blisters; two or three cases I saw terminate in regular tertian intermittent. My patients bore bleeding well; it was however only in cases where inflammation rendered it necessary that I had recourse to it. Convalescents recovered their strength in general very slowly. The progress of the disorder from east to west was so remarkably distinct as not to escape common observation.

14. Dr. Metford, Taunton, April 12, 1803.

I do not think influenza to be contagious.

I have never been able to trace it by infection in a single instance. Though many in a family have had the complaint, it has not attacked those in particular who have attended on a sick person, but invariably those who have been the most exposed to the sudden changes of the atmosphere. (Six men were at work together in a field in this neighbourhood, all of whom were, on the following day, attacked by the influenza.)

The influenza has been attended and followed by greater prostration of strength than generally attends or follows common catarrh. May not this be imputed to the peculiar state of the atmosphere? Few people with whom I have conversed, though labouring under no immediate disease, but but have complained of more than usual relaxation and languor.

I think many instances could not be accurately distinguished from common catarrh, except by the unusual prostration of strength.

The influenza has certainly been attended with greater and more sudden depression than is almost ever seen in common catarrh. A few instances have been attended with apthæ in the throat in the advanced stages of the disorder.

The inhabitants of the country not being in the custom of recurring to the advice of a physician so early as in cities, seldom indeed till the advanced state of the disorder, or considerable danger be suspected, renders it impossible for me to speak with accuracy to dates. I have seen many instances of the peripneumonia notha during the last three months. I believe I am right in stating the greatest number of patients to have occurred between the 20th of March and the 6th of April.

15. Dr. Robinson, Newcastle, Staffordshire, June 20, 1803.

From the few observations I have made upon the nature of the late epidemic catarrh, or influenza, I am inclined to favour the opinion that it was not contagious, but owing to a peculiar state of atmosphere, aided by the different predisposing and exciting causes of the disease. If the influenza had been contagious, would so many in the district attacked have been affected at one and the same time? or, would it have made so gradual a progress through the country as it appears to have done? In a contagious disorder, the offending matter, as in small-pox, measles, typhus, &c. generally exists for some days in the system, before it produces its specific disease; whereas, when more than one in a family has been attacked by the influenza, they have been affected nearly at the same time, at least not more than a few hours intervening; and, in many instances, not more than one or two in pretty numerous families were affected, though the healthy and sick associated promiscuously together as usual. I, for one, associated with two or three families while labouring under the disorder, and without communicating it to any of those families; I think too, it appeared to be more prevalent in airy and high situations than in the low, and to prevail also more amongst people who were much out in the air, especially cially the night air, than amongst those who were more confined within doors.

The disorder, except where it had been neglected, or in old people, has with us been tolerably mild, and in very few instances fatal. It has varied however, in violence, from that of a mild catarrh to a very severe affection of the lungs, attended with proportional fever, cough, head-ach, &c.—In the more severe cases, the sense of languor, debility, and prostration of strength, were greater and more speedily induced, than I have ever recollected to have seen in any other disease; indeed, it was from these latter symptoms only that in the more mild cases you could distinguish this epidemic from a common catarrh. In some instances, when the disorder had been neglected at its commencement, it ran into a typhoid form, and required the same treatment; but if called in within a few hours after its first attack, the administering a brisk calomel cathartic generally checked its progress, insomuch that not any thing more was afterwards required. An emetic was attended with nearly the same advantage, but did not so effectually check the progress of the disorder as a calomel purge. When the disease was fully formed, I principally relied upon the antiphlogistic regimen, with spts. of Mindererus combined with antimonial wine, and with mucilage of g. arabic, tinct. of opium, or elixir paregoric, to alleviate the cough. The local affection of the head and breast were relieved by the application of blisters. In no instance did I venture upon venesection; and in the few instances where I heard it had been had recourse to, it aggravated rather than relieved the complaint. In some persons predisposed to phthisis and affections of the liver, it induced these complaints with a fatal termination in those affected with the former.

16. Dr. Dixon, Whitehaven, June 17, 1803.

1. Such is the similarity in the appearance of the symptoms which occur in catarrh and influenza, that under particular circumstances, it must be extremely difficult to distinguish them. It may however be observed, that the almost universal prevalence of the latter, its frequently assuming the form of a general fever, with more or less of an inflammatory determination to the head, and at the same time, a perfect freedom from any pulmonic disease, constitute the principal difference. In our late epidemic, the effects of the mode of treatment readily discovered the nature of the disease; febrifuge medicines and plentiful dilution lution relieved and recovered the patient, but oleaginous pectorals sensibly aggravated the symptoms.

2. The influenza was first noticed in the month of March. Applications were made for relief at the Dispensary upon the 1st. but it did not engage the attention of private practitioners before the 13th. It was most generally prevalent from the 15th of April to the 1st of May, and did not subside gradually, but almost totally disappeared on or about the 30th.

It began and ended in the adjacent country; in towns or large villages its progress was more rapid. The elevated northern situations in this neighbourhood, remote from each other, first suffered the disease; and soon after it very generally prevailed in Whitehaven. The villages to the south of this town last experienced its influence.

3. From the causes and symptoms of the influenza, the situations it first occupied, and other concomitant circumstances, I am disposed to consider it as a purely inflammatory disease; which has rarely, if ever, been communicated by contagion.

I. Its origin in this country can be ascribed to no other cause than a sudden transition of the atmosphere, from a remarkable degree of heat and moisture to a cold, dry, and windy state.

II. It first appeared, and at the same time, in lofty or exposed northern situations, and these considerably distant from each other.

III. It has been observed that the appearance of purely inflammatory diseases, in this country, is almost invariably preceded, or accompanied, by winds from the north, or north-east, and that persons exposed to these winds are most liable to suffer such diseases. Their origin, therefore, has with justice been imputed to the prevalence of northerly winds. And as during the continuance of the influenza, the wind was generally from the north, this circumstance is a strong confirmation of my opinion, that the disease was of a purely inflammatory nature.

IV. The mode of its appearance was very uncertain. Sometimes it would instantly attack the whole of a family; at others, in irregular succession; but from its progress we could not suspect its contagious power. Frequently only one was affected, and the rest of the family, though constantly attending the patient, escaped the disease.

V. What forms a striking contrast between the influenza and contagious diseases, no predisposition was required for its excitement; and it might have been supposed, that if it were were really of an infectious nature, it would certainly have been communicated to a healthy person, sleeping during the whole course of the disease with one who suffered it in the highest degree; whereas the contrary has happened in several instances.

VI. The symptoms which constituted the disease were, in every respect, inflammatory, affecting chiefly the head and lungs.

VII. The duration of the disease rarely exceeded four days.

VIII. No tendency to malignant putrescency ever took place.

17. Mr. RICE, of East Looe, June 28, 1803.

The influenza began here about the middle of March, and ceased nearly the middle of May.

It appeared first in the country and went westward.

Single instances occur, even now, in this neighbourhood, (June 28, 1803.)

Several were attacked in one family, at one and the same time.

It appeared to be atmospheric.

The symptoms in general, were of the low nervous kind, attended with cough and diarrhœa; a few instances occurred truly inflammatory.

18. Dr. HARRIES, Haverford West, June 27, 1803.

The first person I saw, or heard of being attacked with the influenza, was a labourer about 36 years of age; it was about the 27th of February. He was attacked the day before I saw him, with shiverings and the usual symptoms of pyrexia, accompanied with cough and a fixed pain in the left side; I found him in this state, with a hot skin, frequent pulse, much pain on inspiration, and considerable thirst. As he had been exposed to vicissitudes of heat and cold on the day of attack, and as there were symptoms of local affection, I directed him to be bled, a saline purgative to be administered; and a blister, after a second bleeding, if the blood exhibited a buffy coat, to be applied to the pained part. The appearance of the blood strongly indicated the propriety of the measure, but the man was worse the following day, and the day after; his pulse was as frequent, but it had rather a fluttering feel; his tongue became dry and furred; he was delirious at times, rested ill at night, and the pain in his side was nearly as bad as ever; this was much relieved by a couple of leeches applied plied to the part. He was allowed wine and water, and opiates at night; under which plan, together with the use of the spiritus vitrioli dulcis, as recommended by Dr. Carmichael Smith, in Duncan's Medical Commentaries some years ago, he was in about nine or ten days free from every symptom of fever, but continued very weak for some time after. In this instance, debility came on very rapidly; and the disease had not been noticed before in the town, nor did it occur to me, at the time, to be the Influenza; but I soon heard, that to the northward, from St. David to Fishguard, several families were attacked by it; and I saw some myself in the complaint, in most respects similar in its symptoms to a common catarrh, accompanied with marks of local affection, in others exempt from these. In a few weeks after this, it was general through this town and county. In some families the whole were attacked with it; while in others, in the same street, one or two individuals only suffered; the remainder continuing exempt. The period of duration might perhaps be limited to six weeks, but I think in some instances it appeared a month later. The last under my observation, and only case where it proved fatal, was that of an elderly gentleman; he died about the beginning (the 11th) of May. It prevailed here at the same time we first heard of its being in Bristol and its neighbourhood.

It did appear, in single instances, after ceasing to be general. It did not appear to me to pass from one person to another. In this family, one only out of seven persons was ill, while in the opposite house, most, if not every individual one after the other, some at the same time, felt its effects: this was also the case in other houses. The disease seemed generally accompanied with phlogistic diathesis, and many in the country were bled apparently with advantage; but I did not think it advisable after the instance first mentioned, particularly as where topical affection seemed to prevail it was removed by other means. I found advantage from emetics, but what I chiefly employed was James's powder, which was very effectual.

19. Mr. Jones, Pembroke, June 27, 1803.

From every inquiry I have made, and from what has fallen under my own immediate observation, the epidemic so prevalent through the kingdom, first appeared in this neighbourhood from the last week in March to about the middle or latter end of April; though I had before that time some accidental patients which were attacked with catarrhal fevers, some of short, others of long duration, from about the latter end of January. Those I supposed at the time to be of that genera of fevers which usually appears in this neighbourhood at that season of the year; but, which I am since convinced, was of the same species with that now denominated the Influenza. When it became general here, its progress was so rapid through families, that it scarcely gave time for any infectious effluvia to be communicated, as in the course of two or three weeks there were scarcely one in fifty who escaped more or less an attack. It seemed more fatal to infants under four years, and elderly people who before laboured under any other chronical disorder. They that died were carried off from the fourth to the ninth day, the disorder seeming to attain a degree of crisis on or about the latter day, at which time the general system seemed to shew a strong disposition to putridity. When the epidemic ceased to be general, it occurred frequently in single instances, and that through the greater part of the month of May, even now I witness attacks which manifest a strong tendency towards those of the month of April. One or two instances have occurred, which led me to suppose that the disorder might have been in some degree contagious; as, when a person that had been disordered was shortly removed into a family a little distant from the town, and where no part of the family before had betrayed the least symptom of disorder, it in three or four days broke out. Whether this was to be imputed to contagion or a predisposition in the atmosphere of that neighbourhood, before the removal of the disordered person, is what I cannot give any opinion upon; nor, in any instance but these, can I suppose that the infection had been communicated by contact or in clothes.

20. Mr. Creaser, Bath.

I am not in possession of a sufficient number of data to form a conclusive inference, as my surgical practice is much more extensive than my medical; yet I have seen many cases of influenza. In general, I think that those branches or members of a family which were in the most frequent habits of communication and contact, suffered most; I yet know an instance where, in the case of two sisters sleeping together, one did not receive the disease from the other, although other parts of the family received it. In the decision concerning contagion, more stress should should certainly be laid on positive, than on negative facts.

I think in the general variety, the influenza had but little in common with catarrh. The inflammation of the Schneiderian membrane, and that of the mucous membrane of the bronchiæ were much more frequently absent than present, and when existing did not form the prominent feature as in common catarrh. Symptoms attended which are rarely or never co-existent with catarrh. The usual primary symptoms of typhus and synochus, especially a sudden accession of head ach, with chilliness and prostration of strength, were the first indications. By conversation with some of the most eminent of the London practitioners, I learned, that pains of the articulations, particularly of the ancles and shoulders, were excessive;* sore throat was a common part of the disease. The disorder has often been protracted to the common term of fever, viz. the second or third week.

I have before mentioned the depression of strength and other symptoms as essentially differing in this complaint from the ordinary concomitants of catarrh; yet it is certain, that catarrh, or any other local inflammation, will in some habits, produce febrile symptoms of the typhoid kind. When pneumonia appeared in the influenza, it was connected with a different constitutional affection from that of pure pneumonia.

I saw the first well-marked case at the latter end of February, and the last on the 4th of April.

21. Dr. Sainsbury, Corsham, April 12, 1803.

It is doubtful with me if the present influenza can be attributed to the contagion that arises from one diseased human body pervading and generating it in another.

At a boarding school in this neighbourhood, containing nearly sixty young gentlemen, about fifteen of them had the disease (well marked cases) three weeks since, and the others, notwithstanding some of them were bed-fellows, did not take it.

I believe the influenza is distinguishable from a common catarrh, as, (in many cases) the febrile symptoms commonly run higher, and the depression of strength is more sudden, and much greater.

---

* A lady of fashion, to my knowledge, made a figurative delineation of the disease, in which demons were displayed pinching particular joints.

In some instances the catarrhal symptoms were very slight; but in most cases the languor and debility were considerable, and in some very great, much more than is usually observed in common catarrh.

In one instance the patient, after being first attacked with pneumonia, gradually sunk into typhus, and died. This person had, the week before he was attacked, undergone a chirurgical operation, and was in a very debilitated state.

The first case of well marked influenza that came under my care was (I believe) about the 27th or 28th of February; and the last, on Sunday the 10th of April; but the disease is still prevailing.

22. Dr. Blake, Taunton.

From the few observations I have been able to make, I cannot think the influenza is strictly a contagious disease; and it can only prove so, in my opinion, when (like other febrile disorders) it runs on in peculiar constitutions towards a fatal termination, by producing those symptoms of the greatest debility, known by the name of symptoms of putrescency.

Observations that were general only at first tended to form this opinion; but some particular cases have also had great weight in confirming that opinion.

An old lady, confined to her house by age, and not in any accountable way exposed to contagion, had an attack of influenza, whilst none others of the family had any symptoms of the disease. And some instances have come within my knowledge, in which one of two persons, who slept together in the same bed, and who were almost constantly together during the day, has escaped, whilst the other was suffering from the complaint.

I do not think that any peculiar diagnostic symptoms can be laid down, so as satisfactorily to distinguish the influenza from catarrh.

In various instances, the symptoms seemed to run into each other by almost insensible degrees. In mild cases, therefore, I should call this disease by the former name, and in more violent ones give it the prevailing name of the latter.

I have, at different periods, seen cases, in which great depression has attended an attack of common catarrh; but that affection has without doubt lately shewn itself in a very *remarkable manner* in the influenza. I have also seen cases, in which the other morbid symptoms of influenza have prevailed to a considerable degree, without any very evident state of depression. It appears to me very difficult, in general practice, to affix the bounds of certain disorders, the symptoms of which are so nicely discriminated, and laid down with so much apparent accuracy by nosological writers: and I am very much inclined to think that this disorder may, at various times, have appeared in almost all the gradations from pneumonia to low fever.

The supposition of the influenza being a contagious disease, does not appear to me *of itself* sufficient to account for its rapid and extensive spread; nor do I think an adequate cause can well be assigned, unless it be attributed, in a very especial manner, to some peculiar changes and unknown states of the atmosphere.

23. A Physician, who does not give his Name.

Whether epidemic catarrhs are contagious or not, has been a subject of controversy from the earliest accounts we have of them, and from no observations on the present epidemic have I been enabled to decide the question. Pyrexia, with an unusual degree of languor, lassitude, and general debility in the animal functions, accompanied with head-ach, great confusion of thought, arising often suddenly to delirium, or drowsiness and stupor, appear to be the characteristics of the prevailing disease. Catarrhal or pneumonic symptoms generally attend, or inflammation of some other secreting surface; but I have met several cases without them, and it is not uncommon for the fever to remain after they have vanished. In a few instances, on the second or third day, an eruption like measles has come out. When the disease first appeared in the end of February, the catarrhal symptoms were more frequent and severe; at present the febrile, particularly the affection of the head, are most prevalent and distressing. When the catarrhal affection attends, the disease has been more tedious; when fever alone, it generally runs its course in a few days, frequently leaving lassitude and depression behind for some time. On fresh exposure to cold the cough is readily renewed and also the fever, which in three cases I have observed to assume an intermittent form.

I have met single instances both with and without catarrhal symptoms.

The prevailing epidemic appears to be distinguishable from catarrh, by the suddenness and severity of its attack, by its occurring without exposure to the remote causes of catarrh, catarrh, by the type of the fever, by its continuing after the catarrhal affection is gone, and particularly by its sometimes running its whole course without them. I therefore consider the present disease to be a fever of a peculiar kind, the remote causes of which are highly diffusive; that the catarrhal symptoms are no more essential to it than inflammation of the peritonæum, intestines, or any other secreting surface, but that its being combined with the diseases of the season produced them.

Antimonials, laxatives, and afterwards opium, with blistering, when local inflammation attended, I have found universally successful.

24. *Copy of a Letter to* Mr. Dewar, *Surgeons-Assistant, of the 30th Regiment, from* Mr. Brown, *Surgeon, of Sunderland.*

I would have sent you the information you request, on the subject of influenza, before this time, but could make out nothing satisfactory from the regimental books, as the weekly and monthly returns of the two divisions are so blended together, that it is impossible, at this distance of time, to separate and unravel them. The following is as accurate a statement as can be collected from the hospital and adjutant's books; but it is necessary to premise, that the disease was often so slight, that many of the women and children, as well as of the soldiers, were not confined by it, and of course thought medical assistance superfluous.

The disease appeared about the 21st of February, and entirely subsided by the 23d of March; exactly the period of one lunar revolution. The fine weather introduced along with the new moon, on the last mentioned day, probably produced this salutary change.

The state of the division of the regiment at Sunderland, at the commencement of the epidemic, was nearly as follows.

| | |
|---|---|
| Total strengths, including serjeants, drummers, rank and file - - - - - | 200 |
| Of those who served abroad - - - - - - | 150 |
| Of recruits, joined since landing - - - | 50 |
| Of the old soldiers who took the disease - - | 6 |
| Of the recruits ditto - - - - - - - - - - | 16 |

Out of 20 women who were abroad with the regiment, 5 had the epidemic.

Out of 21 children who were abroad, 6 had the epidemic.

Out

Out of 17 women joined since the return of the regiment, 3 had the epidemic.

Out of 17 children joined, or born, since the return of the regiment, 4 had the epidemic.

For the reasons above mentioned, the number of women and children who had the disease, and even of the soldiers, is far from accurate, as I have only put down those who came under my own observation.

The disease was milder in the barracks than in town; but I only heard of one among the inhabitants who died of it.

The old soldiers had the disease in a slighter degree than the recruits; two or three of the women had it very severely.

All the patients were much relieved by emetics, which produced in all of them copious discharges of bile, and by continuing their operation downwards, or directing it towards the skin, a cure was in most cases obtained.

In one patient the disease was followed by jaundice.

In most of the cases which occurred to me, the catarrhal symptoms were only secondary, and subsided as the bowels were emptied. Slight inflammation of the fauces, accompanied one or two cases. In two instances, where the cough was protracted after the other symptoms had disappeared, the patients were cured by the Peruvian bark. Although the disease, in general, when introduced, went through a whole family, there was only one man in the hospital seized with the epidemic, yet no care was taken to separate the cases admitted with it from the rest of the patients. The men who took the disease in the barracks, were remarked not to belong to one company, or room, in greater numbers than to another, and had often no communication with persons previously affected.

If these remarks can be of any use to Dr. Clark or Dr. Beddoes, they are much at their service; but I am afraid they will go little way to settle the dispute, whether the disease is propagated by human contagion or aerial impregnation.

*May* 20, 1803.

25. *Copy of a Letter from Mr.* Dewar, *Surgeon's Assistant, of the 30th Regiment, to Dr.* Clark, *of Newcastle.*

It was longer than I expected before I received an answer from Mr. Brown, the surgeon of our regiment, otherwise

I should have sent you the statement you wanted much sooner.

TABULAR *Account of the late Epidemic Influenza in Tynemouth Barracks, on a Detachment of the 30th or Cambridgeshire Regiment of Foot.*

| DESCRIPTION of PERSONS. | Numbers who had the Epidemic. | Number who escaped the epidemic | Total. |
|---|---|---|---|
| Serjeants, drummers, and privates, in the barracks, during the prevalence of the epidemic, who had been in foreign service - - - - - - - - - - | 3 | 92 | 95 |
| Ditto in ditto, during ditto, who had joined since the landing of the regiment from the Mediterranean - - - - | 2 | 14 | 16 |
| Women in the barracks, who have been abroad - - - - - - - - - - - - | 2 | 8 | 10 |
| Ditto joined since the landing - - - | 5 | 10 | 15 |
| Children who came with the regiment from abroad - - - - - - - - - | 2 | 8 | 10 |
| Ditto born, or joined, since the landing | 2 | 3 | 5 |
| Officers who have been in foreign service | 1 | 3 | 4 |
| Ditto, who had joined since the landing | 0 | 1 | 1 |
| Total | 17 | 139 | 156 |

The disease was in the barracks of Sunderland exactly one month before it appeared in those of Tynemouth. Mr. Brown remarked to me that, at that time, it prevailed chiefly among the young recruits. This circumstance did not take place in such a striking proportion at Tynemouth. The only patient of mine who suffered much debility, and was confined to bed a number of days, was an old soldier. This was the only patient whose eyes and skin were tinged yellow.

What I have chiefly to remark is, that, from the loose accounts I received of its general prevalence among the inhabitants of North Shields and Tynemouth, where it sometimes pervaded a whole family, it appears to have been comparatively much less general among our soldiers. This circumstance could not have proceeded from the want of exposure exposure to infection, as they had constant intercourse with the people of the neighbourhood, several of them daily frequenting the drinking houses; and those patients who were seized with the epidemic in the barracks were not separated from their companions, especially the women.

The symptoms were much more severe in the women than in the men, excepting in that one man whom I have mentioned.

One query I would start from the statement I have given, and I should wish to see it answered by statements from other regiments. Have soldiers in general, especially old soldiers, been less susceptible than others of this disease? or, has this circumstance been remarked more in some regiments than in others?

Persons who habitually associate together, and are subjected to the same regulations and stile of discipline, certainly receive a train of impressions in common, which may modify the susceptibility of the system, while we will in vain endeavour to particularize the causes. This idea struck me very forcibly in one instance in Egypt. The 24th regiment, which arrived with some others near the close of the campaign, was employed before Alexandria, along with some other regiments, where they were, in every respect, exposed to the same external causes of disease. But that regiment suffered from ophthalmia to a degree unexampled in any other. That disease threatened to render the regiment next to useless, and a considerable number of the men permanently lost their sight, from the traces left by the violence of the inflammation. Within the compass of my knowledge, this was in other regiments a comparatively rare occurrence. All this led me to conclude, that diseases may run in regiments, and in ships, independently of those ascertainable circumstances of external situation that affect the health.

On inquiry of the surgeon of the Lapwing frigate, which has lain for some months in the harbour of N. Shields, I was told that not one man belonging to it had had the epidemic. The surgeon described to me three cases of pulmonic inflammation at that time under his care; but so far as I could judge, they had no appearance of having originated from the Influenza.

You will easily perceive how the circumstances mentioned in Mr. Brown's letter, as limiting the accuracy of his statement, apply to mine.

Perhaps, Dr. Beddoes may think it worth while to request statements from as many surgeons of regiments and of of ships as he can have access to, in order to ascertain the degree in which soldiers and sailors have been susceptible of the disease.

*Tynemouth, May* 24, 1803.

26. Dr. Magennis, Royal Hospital, Plymouth, June 22, 1803.

In conversation with our friend, Mr. Allen, he spoke of your laudable intention of amassing such a body of evidence as would in all probability determine the real nature of the late general catarrhal affection; that is, whether the disease was to be deemed contagious or otherwise. I then mentioned to him the only fact which came to my knowledge, that could at all disturb the opinion I had formed of its non-infectious nature. The circumstance alluded to, occurred in a gentleman's family with whom I am on terms of intimacy, and was as follows. The man servant was first attacked with severe catarrhal symptoms; the complaint continuing to increase in violence, and being a married man, it was judged necessary that he should go home to be nursed by his wife: the cook was then seized, and likewise obliged to quit her service; the woman who succeeded the cook, as a temporary substitute, was in less than forty-eight hours also attacked with similar symptoms, and shortly after the gentleman's daughter. In the mean time the man servant recovered, and returned to duty; but he had not been many days in the house when he was again seized, from which he recovered very slowly, and has continued ever since in a very debilitated state, notwithstanding the use of the most powerful tonics.

It must be confessed that these facts strongly support the contagious nature of the disease; but they are isolated facts as far as my own experience extends, while the tenour of my practice warrants me in drawing a directly contrary inference: but at the same time it must in justice be observed, that, that practice has been, with respect to influenza, extremely limited.

That the influenza travelled from east westerly must be admitted, as the disease existed in its full force many weeks in London, previous to its appearance in the milder climate of the west of England; and it was extremely prevalent in and about Exeter before its influence was at all felt at Plymouth and its environs; where, all of a sudden, it became general throughout the three towns of Plymouth, Plymouth Dock, and Stonehouse, extending in every direction rection to the adjoining country, and so on to the Land's End; the disorder arriving at its acme in a few days from its first appearance.

Does not this sudden and general appearance of the disease militate against the idea of any specific contagion? Are there any well authenticated historical records of contagion, however malignant and destructive, having spread with such celerity? Are its effects as contagion, admitting it such, commensurate to its rapidity and universality? And ought it not rather to be imputed to a peculiar state of the atmosphere? This peculiar constitution of the air in great measure, superinduced by frequent alternating changes from heat to cold, from frost to rain, joined to the unusual prevalence of easterly winds during the winter and commencement of the spring: I say, in great measure, as there is probably a certain something, quality or substance, indefinable, because hitherto it has eluded, and will perhaps for ever elude, the most vigorous efforts of human industry, independent of the usually observed physical properties of air, its various changes and modifications, periodically superadded to the atmosphere of certain portions of the globe, rapidly diffusible, but limited in its extent, course, and duration.

It is however a certain truth, that the ships of war fitted out and manned in the river Thames, had their crews very generally affected with influenza, especially the Albion and Thunderer; both ships having lost a great many hands by that disease. On the Albion's arrival at Plymouth, eight of her men were sent under my care to the Royal Hospital in the most deplorable condition: two died a few hours after admission; but the others recovered, contrary to all expectation. The surgeon, deceived by the severity of the cough, dyspnœa, and stricture on the chest, was at first induced to employ the lancet freely; but he soon perceived that all those who were blooded, sunk speedily after the operation. Two died the day before the ship arrived here, although the disease had existed only six or seven days altogether on board. The complaint continued to spread to an alarming degree for many days after the ship anchored at Cawsand Bay, and proved fatal to several. The Thunderer was equally affected, and about the same period, early in April. I must observe, that nothing like contagion was propagated to the other patients in the ward (being ten in number besides two female nurses) into which the Albion's people were put, although these men were in the last and most aggravated stage of the malady.

One

One of my medical friends states as follows: "About this period (end of March) I was called to the assistance of a few families in Dunford Street; in three of these, the servants were first seized, afterwards the remainder of the family without exception. In two others, the children were seized next to the servants; but the parents escaped; and in one family, a younger daughter conceived she caught the disease by sleeping with her eldest sister."

Whatever may have been the specific nature of the disease, I am persuaded it had lost much of its virulence before it reached us, if I were to draw the inference from a comparison of the mortality caused by it here and to the eastward; for there are but few instances of its having proved fatal in or about this place.

Independent of the numerous deaths caused by the immediate operation of this epidemic, considered simply as such, I have reason to believe that it has been productive collaterally of more extensive mischiefs, by adding largely to the list of consumptive patients. Phthisis has been more than usually prevalent and destructive during the last few months, owing, I think, to the baneful effects of influenza. All those, who from an hereditary constitutional taint, and those predisposed to the disorder from a peculiar idiosyncracy and comformation of system, or those who had already laboured under the slightest degree of pulmonic tubercular irritation, if attacked by the prevailing epidemic, will be found, I fear, ultimately to have fallen victims to pulmonary consumption.

Whether the above remark is likely to correspond with the past experience and future observations of others, I know not; but the fact has forcibly impressed itself on my mind; and, unhappily, I have already witnessed too many cases to strengthen and justify the opinion.

*Q.* When did the influenza begin and cease with you?

*A.* In or about the 20th of March, and wholly disappeared about the beginning of May, or at the latest the middle of that month.

Q. Did it begin and end sooner or later in the country than in the town?

*A.* It shewed itself first in the country, east of Plymouth, and appeared in Cornwall, lying west, later than at Plymouth.

Q. Do you believe it passes from person to person?

*A.* Out of seven medical men of extensive practice consulted, four gives an opinion of its non-contagious nature, two two are doubtful, rather inclining to its being infectious, and the other decides it contagious.

It is difficult to account for the various dissimilar and contradictory opinions which the medical world entertain respecting the nature of this malady. I feel great pleasure in communicating to you every fact that came under my own observation, as well as those which I have been able to collect from others, and I lament only that their paucity should render them of so little value.

27. Dr. Bree, Birmingham, April 10, 1803.

I have not been able to satisfy myself on this head. A person came from London with the epidemic upon him. As he had travelled all night, and was an old asthmatic, he did not at first consider his disorder as the influenza, which it clearly was.

This patient remained in a family under every symptom of the influenza, till he could pursue his journey, twelve miles further. None of the family, in which he staid a week, were infected by him.

The epidemic is distinguishable from a common catarrh by the loss of strength and degree of fever, and more particularly by the slow progress to recovery, and the great depression of mind which remains till the strength is restored.

In single instances the disease was frequently very mild; and where it went through families, it was frequently so mild as not to be distinguished from a common catarrh; it would therefore be properly termed a catarrh in such instances, the loss of strength and degree of fever being not considerable; yet there might be observed more depression in convalescence than usually follows or accompanies the progress of common catarrh.

In some few instances the disorder was attended by prostration of strength, equal to that of low fever; but in these, evacuations had been too large, or too long continued.

No case well marked occurred to me before the 6th of March: The disease has not yet finished its course here.

28. Dr. Alderson, Hull.

The influenza made its appearance here after some warm weather in the beginning of March, which was suddenly succeeded by a strong and very cold south-west wind, veering to the northward and loaded with sleet. At this time, as as might have been expected, several catarrhs and some pneumonias made their appearance; but as reports of similar affections, with some distinctive marks, were prevalent in other parts of the country, we had no doubt in classing the epidemic under the fashionable title. Its most characteristic marks were fever, head-ach, weight on the eyes, pain in the arms and limbs, but the aching in the arms was the most pointed symptom. In some the irritation was nearly membraneous; in others, the stomach and biliary secretions were the seat of disease; in all, considerable fever: and as it began at different times in different parts of the country, there was reason to suppose it contagious; but all who got cold at that period, and for six week afterwards, had nearly similar symptoms. In no family that I attended were all affected; but in general it has been so slight a disease, that I have scarcely ever prescribed for more than one in a family. Amongst the lower orders, from inattention to the inflammation along the mucous membrane extending down the bronchia, I can perceive symptoms of phthisis. Of these I have seen above a hundred, who began their tale with, "Sir, about six weeks ago I had the complaint, but thought nothing of it at the time."

The present epidemic is the inflammatory sore throat; several of them have run into abscesses, and in one instance I think the inflammation had extended into the trachea; for when it broke, suffocation was induced by the quantity of matter discharged. Where the stomach was the principal seat, an emetic, followed by rhubarb and salt of tartar, cured. Where the chest was affected, the blood was commonly sizy; and such were all benefited by one bleeding.

I have found the bark and acids hurtful, the extract of poppies the most useful sedative, and the common spirit of Mindererus, the most generally useful febrifuge.

29. Dr. Blount, Hereford, June 28, 1803.

The influenza appeared in these parts soon after Christmas, became very general in February and March, and began to disappear in April; but solitary instances of it were to be found till the middle of this month.

The first cases of it that occurred to me, were remote in the country, and among persons that did not seem to have had any connection with any one affected with it.

It often seemed to pass from person to person in the same family, but by no means universally so, for children generally generally escaped, particularly young children, two only out of eight, in my own family, had it very slightly. The lunatics in our asylum were not affected by it. The old, the feeble, and particularly the asthmatic, were severely treated by it, but to few, very few indeed, did it prove fatal in these parts.

It did not appear to me to be conveyed by clothes or other instruments, but to be propagated by the atmosphere.

[ To be continued. ]

